# Green Synthesis and Flexibilization Engineering of (ECMP)_2_MnBr_4_ for Smart Textile‐Integrated Luminescence

**DOI:** 10.1002/advs.202511652

**Published:** 2025-08-31

**Authors:** Xiao Wang, Yanyan Li, Haitao Tang, Wenxuan Yao, Jiao Li, Fang Yao, Weibing Zhong, Kangyu Jia, Dong Tu, Qianqian Lin, Mufang Li, Dong Wang

**Affiliations:** ^1^ Key Laboratory of Textile Fiber and Products Wuhan Textile University Ministry of Education Wuhan 430200 China; ^2^ School of Physics and Technology Wuhan University Wuhan 430200 China; ^3^ Faculty of Materials Science and Chemistry China University of Geosciences Wuhan 430074 China

**Keywords:** 0D perovskites, green synthesis, manganese bromides, smart textiles

## Abstract

0D hybrid manganese halides represent an emerging class of luminescent materials, yet their practical application has been hindered by the intrinsic trade‐off between optical performance and mechanical flexibility. Here, a green synthesis of 0D (ECMP)_2_MnBr_4_ crystal is reported, exhibiting unprecedented triple‐mode emission (photoluminescence, X‐ray scintillation, and mechanoluminescence) through rationally designed highly symmetric [MnBr_4_]^2−^ tetrahedra, achieving near‐unity photoluminescence quantum yield (98.97%), record‐low X‐ray detection limit (15.62 nGy_air_ s^−1^) and multi‐stimuli responsiveness (rubbing, squeezing, stretching). The material's ultralow electron‐phonon coupling (*S* = 1.438) and defect‐suppressing *π–π* stacking enable exceptional environmental stability and closed‐loop recyclability via solvent‐mediated recrystallization. Innovatively, (ECMP)_2_MnBr_4_ is first integrated into thermoplastic polyurethane via wet‐spinning, simultaneously retaining single‐crystal emission intensity and achieving remarkable elasticity (>1000% strain) for deformation‐resistant wearable applications. This work establishes a new design paradigm for sustainable multifunctional optoelectronics, with immediate applications in wearable displays, high‐resolution X‐ray imaging, and self‐powered optical sensors.

## Graphical abstract

Green‐synthesized (ECMP)_2_MnBr_4_ achieves near‐unity photoluminescence quantum yield (98.97%) and triple‐mode emission (photo/X‐ray/mechano), enabling wearable anti‐counterfeiting textiles, ultrasensitive X‐ray imaging (15.62 nGy_air_ s^−1^), and demonstrating potential for optical stress sensing. Scalable wet‐spun fibers ensure mechanical robustness and ambient processability.

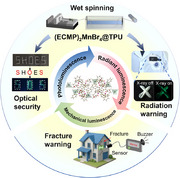

## 1 Introduction

In recent years, halide perovskites have emerged as promising materials for optoelectronic and light‐emitting applications due to their tunable optical bandgaps, exceptional optoelectronic properties, and outstanding charge‐carrier transport characteristics.^[^ ^1^, ^2^, ^3^ ^]^ However, their commercial adoption faces three fundamental challenges: i) intrinsic instability stemming from their ionic nature, which is extremely sensitive to environmental factors such as moisture, temperature, and illumination; ii) environmental and biosafety concerns associated with lead toxicity; and iii) nonradiative recombination losses caused by high defect densities, which typically limit their photoluminescence quantum yield (PLQY) below the theoretical threshold of 90%.^[^ ^4^, ^5^, ^6^ ^]^ These critical issues severely constrain the practical application and operational lifetime of perovskite‐based devices, creating an urgent need for environmentally benign alternatives.

Recently, 0D organic–inorganic hybrid metal halides have attracted considerable attention owing to their unique structural characteristics. These materials feature isolated [MX_6_]^4−^ octahedral or [MX_4_]^2−^ tetrahedron units spatially separated by bulky organic cations, resulting in pronounced quantum confinement effects.^[^ ^7^, ^8^ ^]^ This distinctive architecture endows them with exceptional photophysical properties, including strong exciton binding energies (> 170 meV for Cs_4_PbI_6_),^[^ ^9^ ^]^ high defect tolerance, and remarkable luminescence efficiency.^[^ ^10^, ^11^ ^]^ Precise control over their optical characteristics‐including emission wavelength, full‐width at half‐maximum (FWHM), and PLQY can be achieved through strategic selection of metal centers (e.g., Mn^2+^, Sn^2+^, Sb^3+^), halide ligands (e.g., Cl^−^, Br^−^, I^−^) and organic cations (e.g., Tetrapropylammonium cation (TP^+^), Triphenylsulfonium cation (TPS^+^), Benzyltriphenylphosphonium cation (BTP^+^), Ethyltriphenylphosphonium cation (ETP^+^), Hexyltriphenylphosphonium cation (HTP^+^)), making them highly promising for optoelectronic applications.^[^ ^12^, ^13^, ^14^ ^]^

Despite their remarkable optoelectronic properties, the practical implementation of 0D metal halides faces several challenges in both material synthesis and application. First, conventional synthesis methods typically require high‐boiling‐point toxic solvents (*e.g*., N, N‐Dimethylformamide (DMF), acetonitrile), high‐temperature processing (> 240 °C), or complex antisolvent diffusion techniques. For instance, Han et al. synthesized TPP_2_MnBr_4_ single crystals by dissolving tetraphenylphosphonium manganese halide (TPPBr^+^: MnBr_2_ = 2:1) in DMF, followed by ether vapor diffusion.^[^ ^15^ ^]^ Li et al. required 240 °C to prepare bis(4‐methylthiophenyl)phosphonium tetrabromomanganate(II) ((4‐MTPP)_2_MnBr_4_) glass.^[^ ^16^ ^]^ Wang et al. used a methanol/concentrated HBr mixed solvent system to grow (ETP)_2_MnBr_4_ single crystals.^[^ ^17^ ^]^ Gong et al. synthesized bis(benzyltriphenylphosphonium) tetrabromomanganate(II) ((BzTPP)_2_MnBr_4_) scintillator crystals using ethanol/acetonitrile mixed solvents.^[^ ^18^ ^]^ These approaches not only involve complex procedures and high energy consumption but also pose environmental risks. Second, most reported Mn‐based halides exhibit only single‐ (e.g., photo‐ and X‐ray‐responsive) or dual‐mode (e.g., simultaneous response to light and X‐rays) luminescence,^[^ ^19^, ^20^, ^21^ ^]^ falling short of multifunctional integration requirements. More critically, materials with narrowband emission often sacrifice mechanical flexibility. Furthermore, the inherent contradiction between conventional high‐temperature solid‐phase synthesis (> 300 °C) and the low‐temperature processing requirements of flexible electronics severely limits their applications in emerging fields like wearable devices.

To address these challenges, we propose an innovative “green synthesis coupled with crystal rigidification‐flexibilization” strategy. At the molecular design level, we selected ethoxycarbonylmethyl triphenylphosphonium (ECMP^+^) as the organic cation, where the phenyl rings enhance crystal rigidity (suppressing nonradiative transitions and boosting luminescence efficiency) through *π–π* stacking, while the flexible ester substituent imparts deformability. For synthesis, we selected an environmentally benign, low‐cost solvent evaporation method at 70 °C, enabling one‐step preparation of high‐purity (ECMP)_2_MnBr_4_ single crystals from ethanol solutions containing ethoxycarbonylmethyl)triphenylphosphonium bromide and MnBr_2_‐completely avoiding toxic solvents and high‐temperature processing. Remarkably, the resulting crystals demonstrate tri‐mode luminescence (photo‐, X‐ray‐, and mechano‐luminescence) with excellent environmental stability and recyclability. Subsequently, through wet‐spinning integration with thermoplastic polyurethane, we successfully fabricated highly stretchable and stable luminescent fibers. Interestingly, the (ECMP)_2_MnBr_4_@TPU composite fibers maintain 90% of initial luminescence intensity under 100% strain, overcoming the flexibility limitations of conventional luminescent crystals and enabling innovative applications in smart textiles, including advanced anti‐counterfeiting systems and wearable X‐ray dosimeters with real‐time monitoring capabilities.

## 2 Results and Discussion

**Figure 1:**
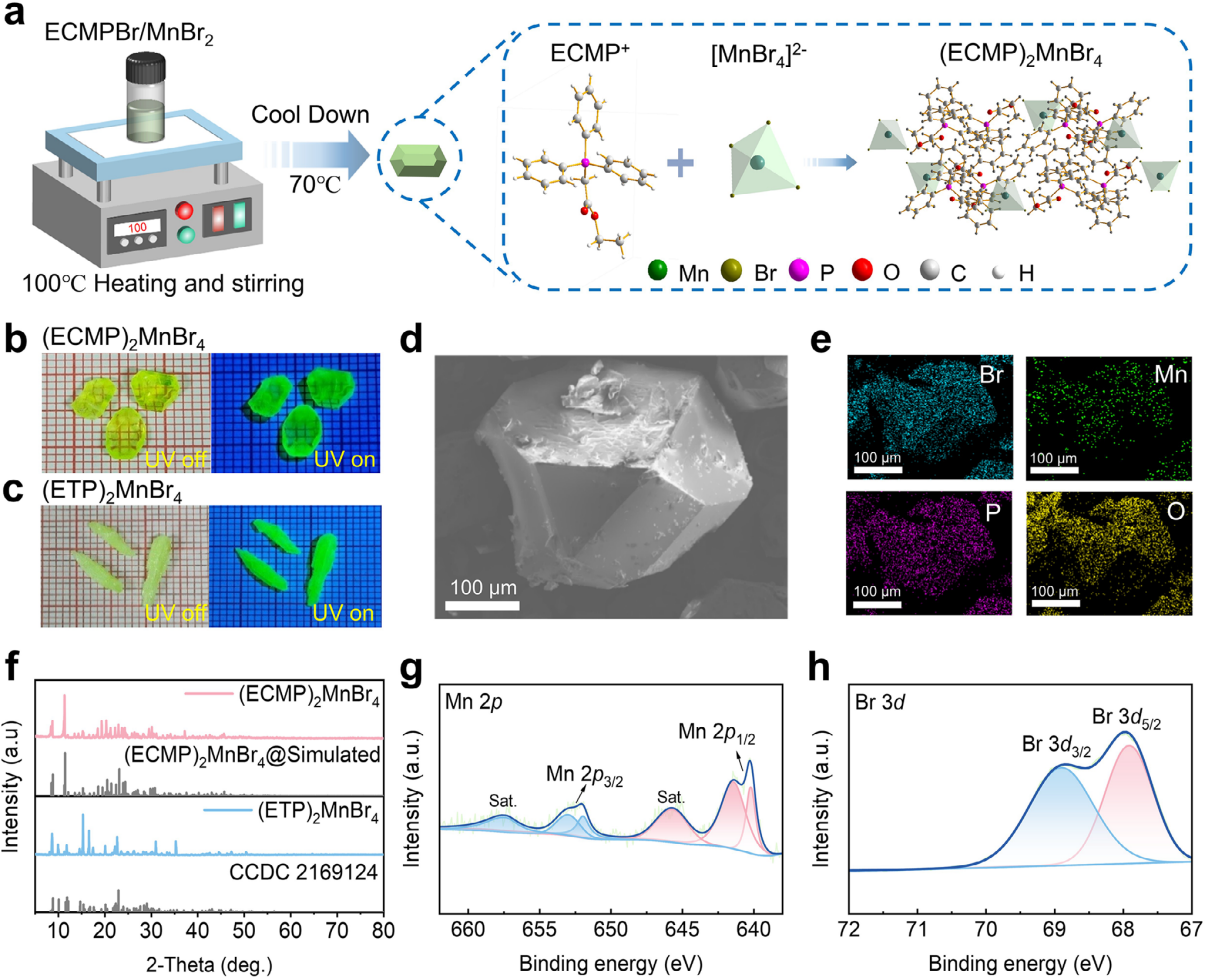
a) Schematic illustration of the solvent evaporation synthesis and corresponding crystal structure of (ECMP)_2_MnBr_4_, (b,c) Optical microscopy images showing the macroscopic morphology of (ECMP)_2_MnBr_4_ and (ETP)_2_MnBr_4_ crystals under white light and 365 nm UV illumination (scale bar: 1 mm per grid division), d) SEM image and e) Corresponding EDS elemental mapping of (ECMP)_2_MnBr_4_, f) XRD patterns of both (ECMP)_2_MnBr_4_ and (ETP)_2_MnBr_4_ crystals, XPS spectra of (ECMP)_2_MnBr_4_, g) Mn 2*p* and h) Br 3*d* regions.

High‐quality (ECMP)_2_MnBr_4_ single crystals were successfully synthesized via a facile solvent evaporation method. Specifically, ethoxycarbonylmethyl)triphenylphosphonium bromide (ECMPBr)photoluminescence (and manganese(II) bromide (MnBr_2_) powders were dissolved in 5 mL of ethanol at a molar ratio of 2:1. After complete dissolution under stirring at 100 °C, the solution was maintained at 70 °C for solvent evaporation, yielding well‐formed single crystals. The crystal structure of (ECMP)_2_MnBr_4_, as determined by single‐crystal X‐ray diffraction, is presented in **Figure** **1**a (Crystal structure parameters, bond lengths, and angles are summarized in Tables S1–S3, Supporting Information). The longest and shortest distances between adjacent Mn^2+^ ions are 13.167 and 10.129 Å in (ECMP)_2_MnBr_4_, respectively (Figure S1, Supporting Information), significantly exceeding those reported for conventional organic–inorganic manganese halides previously reported and are conducive to the photoluminescence (PL) efficiency. For comparative analysis, (ETP)_2_MnBr_4_ crystals were synthesized under identical conditions as our compound, serving as an ideal reference material due to their structural homology (sharing [MnBr_4_]^2−^ tetrahedra while differing only in phosphonium cations, ETP^+^ vs ECMP^+^) and well‐documented photophysical properties in manganese‐based hybrid halides. Optical microscopy observations (Figure 1b,c) revealed that both crystal types exhibited polyhedral morphologies with centimeter‐scale dimensions, emitting intense green emission under 365 nm UV excitation. Significantly, the (ECMP)_2_MnBr_4_ crystals displayed superior morphological regularity and optical transparency relative to its (ETP)_2_MnBr_4_ counterpart. Figure S2 (Supporting Information) illustrates the size distributions of 45 single crystals of (ECMP)_2_MnBr_4_, showing an average crystal size close to 4 mm, marginally smaller than that of (ETP)_2_MnBr_4_. Scanning electron microscopy (SEM) characterization (Figure 1d) confirmed the defect‐free surfaces in (ECMP)_2_MnBr_4_ crystals, contrasting with the more irregular morphology observed in (ETP)_2_MnBr_4_ (Figure S3, Supporting Information). Energy‐dispersive X‐ray spectroscopy (EDS) elemental mapping in Figure 1e verified the homogeneous distribution of Br, Mn, P, and O throughout the crystal lattice, confirming excellent compositional uniformity. X‐ray diffraction (XRD) patterns (Figure 1f) matched perfectly with the data in the standard database, confirming no impurity phases. High‐resolution X‐ray photoelectron spectroscopy (XPS) provided atomic‐level resolution of the chemical environment (Figure 1g,h; Figure S4, Supporting Information). The Mn 2*p* spectrum exhibited characteristic peaks at 641 eV (2*p*_3/2_) and 653 eV (2*p*_1/2_), confirming the presence of Mn^2+^. The Br 3d spectrum displayed symmetric doublets at 68 eV (3*d*_5/2_) and 69 eV (3*d*_3/2_), consistent with Br^−^ ligands in [MnBr_4_]^2−^ units. The absence of peak splitting indicates uniform coordination environments for all bromine atoms. The comprehensive characterization reveals three critical structural advantages of (ECMP)_2_MnBr_4_, highly symmetric [MnBr_4_]^2−^ coordination units, uniform chemical environments, and strong Mn‐Br coordination bonds. The structural perfection directly correlates with the exceptional PL properties, including both high emission intensity and quantum yield (PLQY). For comparative analysis, XPS analysis of (ETP)_2_MnBr_4_ (Figure S5, Supporting Information) reveals analogous P 2*p*, Mn 2*p*, and Br 3*d* signatures, confirming the preservation of the [MnBr_4_]^2−^ framework. However, subtle shifts in binding energies (≈0.3–0.5 eV) suggest minor differences in ligand‐field effects between the two compounds, likely arising from distinct organic cation environments. To investigate the thermal stability of (ECMP)_2_MnBr_4_, thermogravimetric analysis (TGA) and differential scanning calorimetry (DSC) measurements were performed. The TGA results demonstrate that (ECMP)_2_MnBr_4_ exhibits excellent thermal stability below 200 °C, with no significant weight loss indicative of decomposition (Figure S6a, Supporting Information). DSC analysis reveals a distinct endothermic peak at 155.49 °C, corresponding to the melting transition of the compound (Figure S6b, Supporting Information).

**Figure 2:**
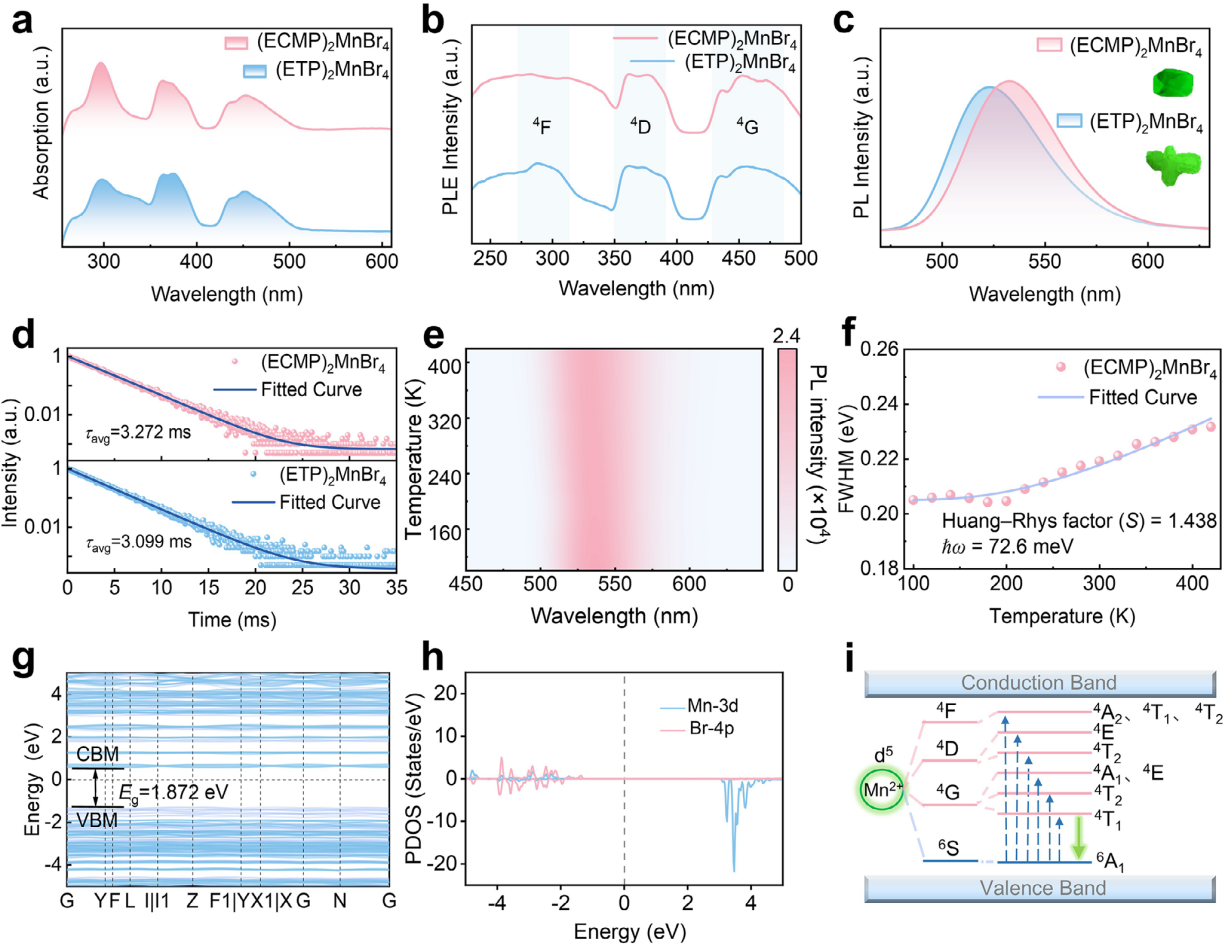
a) UV–vis absorption spectra, b) PLE spectra, c) PL emission spectra under 365 nm excitation, d) Time‐resolved PL decay curves of (ECMP)_2_MnBr_4_ (up) and (ETP)_2_MnBr_4_ (down) with biexponential fitting, e) Temperature‐dependent PL spectra of (ECMP)_2_MnBr_4_ measured from 100 to 420 K with 20 K intervals, f) Temperature‐dependent FWHM of the (ECMP)_2_MnBr_4_ (pink symbols) PL spectra and the corresponding theoretical fit (blue curve), g) Calculated electronic band structure of (ECMP)_2_MnBr_4_, h) Corresponding projected density of states, i) Energy level diagram depicting the electronic transitions and relaxation pathways in (ECMP)_2_MnBr_4_, including the parity‐forbidden ^4^T₁→^6^A₁ transition responsible for green emission.

Following thorough structural characterization, we systematically investigated the optical properties of (ECMP)_2_MnBr_4_ and (ETP)_2_MnBr_4_ single crystals. UV–vis absorption spectroscopy (**Figure** **2**a) revealed three characteristic absorption peaks between 250–550 nm for both compounds, (ECMP)_2_MnBr_4_ exhibiting slightly higher absorption intensity than (ETP)_2_MnBr_4_, indicating superior light‐harvesting capability. Photoluminescence excitation (PLE) spectra (Figure 2b) showed nearly identical peak positions for both crystals, suggesting similar electronic transition processes. Detailed emission studies demonstrated that (ECMP)_2_MnBr_4_ maintains narrowband green emission centered at 530 nm (FWHM = 55 ± 2 nm) across all excitation wavelengths (Figure S7, Supporting Information). Notably, (ECMP)_2_MnBr_4_ exhibits significantly higher PL intensity than (ETP)_2_MnBr_4_ in Figure 2c, attributable to its more ordered crystal structure that effectively minimizes lattice defects‐known non‐radiative recombination centers that dissipate exciton energy through thermal or phonon pathways. Quantum yield measurements (Figure S8, Supporting Information) quantified this performance advantage, with (ECMP)_2_MnBr_4_ achieving a near‐unity PLQY of 98.97% compared to 87.21% value for (ETP)_2_MnBr_4_. This 11.76% absolute improvement underscores the critical role of ECMP⁺ ligands in optimizing the Mn^2+^ coordination environment for maximum emission efficiency. Figure 2d shows the time‐resolved photoluminescence spectrum, and the decay curves can be well fitted by biexponential decay kinetics,

(1) $$\begin{array}{c} I\left(t\right)=A_{1}\;e^{-\frac{t}{\tau_{1}}}+A_{2}e^{-\frac{t}{\tau_{2}}} \end{array}$$

where *I* (*t*) represents the time‐dependent PL intensity, *t* denotes the decay time, *τ*_1_ and *τ*_2_ correspond to the characteristic lifetimes of the fast and slow decay components, respectively, and *A*_1_ and *A*_2_ are the relative amplitudes of each decay pathway. Based on the *τ*_1_ and *τ*_2_ values, the average lifetime (τ) can be calculated by,

(2) $$\tau =\frac{A_{1}\tau_{1}^{2}+A_{2}\tau_{2}^{2}}{A_{1}\tau_{1}+A_{2}\tau_{2}}$$

(ECMP)_2_MnBr_4_ and (ETP)_2_MnBr_4_ exhibit comparable fluorescence lifetimes of 3.272 and 3.099 ms, respectively, indicating that ligand modification primarily affects radiative recombination efficiency rather than excited‐state lifetime. The complete biexponential fitting parameters (*τ*_1_, *τ*_2_) are systematically presented in Table S4 (Supporting Information).

To elucidate the origin of the exceptional performance of (ECMP)_2_MnBr_4_, we conducted temperature‐dependent PL studies from 100–420 K (20 K intervals, Figure 2e). The emission peak position remains remarkably stable with temperature variation, demonstrating exceptional rigidity in the Mn^2+^ coordination environment. Thermal quenching, a universal phenomenon in luminescent materials caused by electron‐phonon coupling‐induced non‐radiative relaxation,^[^ ^22^, ^23^ ^]^ was quantitatively analyzed through the Huang‐Rhys factor (*S*) and activation energy (*E*_a_). The *S* serves as a critical indicator of electron‐phonon coupling strength, where *S*≈1 representing weak coupling while *S* > 5 reflects strong coupling.^[^ ^24^ ^]^ *S* can be obtained through fitting the temperature‐dependent FWHM of the emission spectra (Figure 2f; Figure S9a, Supporting Information) with the following equation,^[^ ^25^ ^]^

(3) $$\mathrm{FWHM}\left(T\right)=\sqrt{8ln2}\hbar \omega \sqrt{\mathrm{Scoth}\left(\frac{\hbar \omega }{2k_{B}T}\right)}$$

where ℏω, *k*_B_, and *T* are the phonon energy, Boltzmann constant, and absolute temperature, respectively.

The best fitted result with *R* ^2^ = 0.98 was obtained with *S* = 1.438 and ℏω = 72.6 meV. The significantly lower Huang‐Rhys factor (*S* = 1.438) compared to commercial phosphors like YAG:Ce^3+^ (*S* = 6)^[^ ^23^ ^]^ indicates reduced lattice distortion during excitation, demonstrating substantially weaker electron‐phonon coupling in the(ECMP)_2_MnBr_4_ system. Furthermore, we quantified the thermal quenching behavior through Arrhenius analysis (Figure S9b, Supporting Information), where the *E*_a_ serves as a direct measure of the luminescent center's thermal stability. A higher *E*_a_ value indicates greater resistance to thermal quenching, providing crucial guidance for material optimization. The temperature‐dependent luminescence intensity was fitted using the following Arrhenius relation,^[^ ^26^ ^]^

(4) $$I\left(T\right)=\frac{I_{0}}{1+\mathrm{Aexp}\left(-\frac{E_{a}}{k_{B}T}\right)}$$

where *I*_0_ is the integrated PL intensity when the temperature at 0 K, *k*_B_ is the Boltzmann constant.

Arrhenius analysis yields an *E*_a_ of 188.7 meV, reflecting strong thermal quenching resistance. These parameters collectively explain the material's ability to maintain high emission efficiency at elevated temperatures. The electronic structure of (ECMP)_2_MnBr_4_ was systematically investigated through density functional theory calculations (Note S1, Supporting Information). As illustrated in Figure 2g, the calculated band structure exhibits a direct bandgap of 1.87 eV, consistent with its green emission properties. The projected density of states (PDOS) analysis (Figure 2h) reveals that the valence band maximum (VBM) is predominantly contributed by Mn d‐orbitals, while the conduction band minimum (CBM) is primarily governed by hybridized states of Br p‐orbitals and Mn d‐orbitals. The substantial overlap between Mn and Br PDOS indicates significant covalent interactions in the Mn‐Br bonds. This electronic configuration underscores the pivotal role of the [MnBr_4_]^2−^ tetrahedral units in determining the optoelectronic properties of the material. Furthermore, charge density distribution (Figure S10, Supporting Information) demonstrates spatial localization of CBM and VBM states around Mn and Br atoms, respectively. The CBM exhibits tetrahedrally symmetric electron density around Mn centers, whereas the VBM displays anisotropic contributions from Br ligand p‐orbitals. This delocalized yet coordinated charge distribution facilitates efficient exciton formation and radiative recombination, corroborating the observed high PLQY. The energy level diagram (Figure 2i) illustrates the emission mechanism of Mn^2+^, UV excitation promotes electrons from the ^6^A_1_ ground state to the ^4^T_1_ excited state, followed by spin‐forbidden ^4^T_1_→^6^A_1_ transition yielding efficient green emission. The synergy between this unique electronic transition characteristic and the rigid crystal structure constitutes the fundamental origin of the material's exceptional luminescent properties.

**Figure 3:**
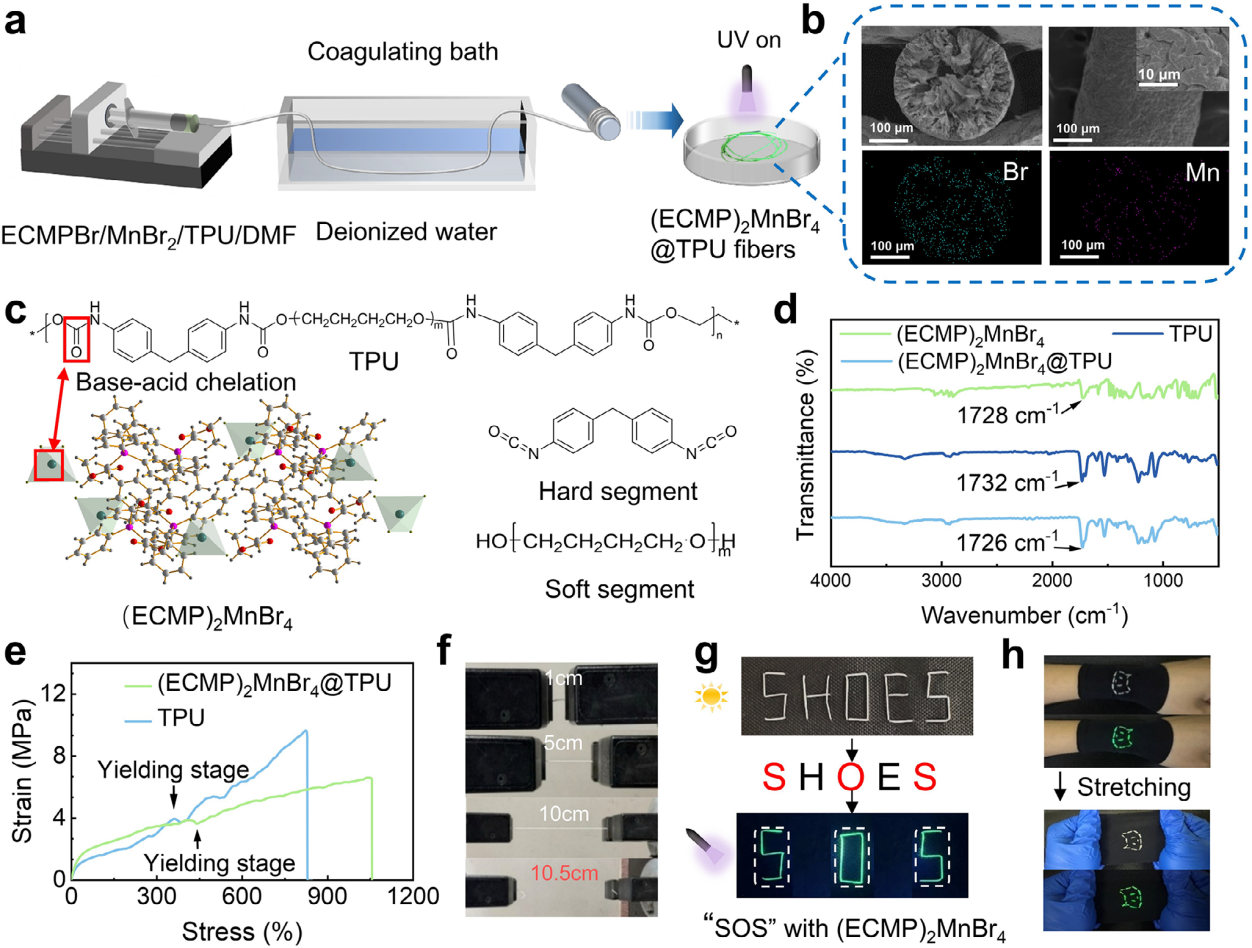
a) Schematic illustration of the room‐temperature wet‐spinning fabrication process, b) Cross‐sectional SEM morphology and corresponding EDS elemental mapping demonstrating uniform distribution of (ECMP)_2_MnBr_4_ within the TPU, c) Molecular interaction mechanism between (ECMP)_2_MnBr_4_ and TPU polymer chains, d) Comparative FTIR spectra identifying characteristic vibrational modes of pristine (ECMP)_2_MnBr_4_, TPU and (ECMP)_2_MnBr_4_@TPU, e) Mechanical performance evaluation through stress–strain curves comparing composite (ECMP)_2_MnBr_4_@TPU fibers and pristine TPU fibers, f) Schematic representation of the mechanical property testing for the luminescent fibers, g) Demonstration of (ECMP)_2_MnBr_4_@TPU fibers for anti‐counterfeiting and encryption applications. h) Stability testing of PL performance under mechanical tensile deformation.

To achieve a breakthrough in wearable optoelectronic applications of 0D organic–inorganic hybrid manganese halide luminescent materials, we innovatively employed a wet‐spinning technique for fabricating (ECMP)_2_MnBr_4_@ thermoplastic polyurethane (TPU) composite fibers that simultaneously exhibit exceptional flexibility and stable luminescence. As illustrated in **Figure** **3**a, the entire fabrication process was conducted at ambient temperature, homogeneous spinning solution was prepared by dissolving ECMPBr, MnBr_2_, and TPU granules in N, N‐dimethylformamide, followed by coagulation in a water bath, luminescent fibers only need to be naturally dried without post‐annealing. Notably, this is the first report of luminescent fibers based on 0D organic–inorganic hybrid manganese halides into a flexible fiber via wet spinning. SEM analysis (Figure 3b) reveals that the obtained fibers possess a continuous porous architecture with uniform surface and cross‐sectional morphology. EDS elemental mapping confirms the uniform distribution of Mn and Br elements throughout the TPU matrix, ensuring consistent optical performance. Remarkably, the porous TPU framework not only maintains the material's high flexibility but also provides an ideal dispersion medium for the (ECMP)_2_MnBr_4_ luminescent centers. The mechanical performance of the composite fibers is fundamentally governed by interfacial interactions between the hard and soft segments of the constituent materials. To evaluate whether the incorporation of (ECMP)_2_MnB_4_ crystals into the TPU matrix affects their luminescent properties, we conducted PL measurements. As shown in Figure S11 (Supporting Information), the PL spectra of pristine (ECMP)_2_MnB_4_ crystals and (ECMP)_2_MnB_4_@TPU composite fibers exhibit nearly identical emission intensities, confirming that the luminescence capability of (ECMP)_2_MnB_4_ remains uncompromised upon integration into the polymer host. In addition, our wet‐spinning process enables the (ECMP)_2_MnBr_4_ crystals to become molecularly embedded within the TPU matrix, creating a dynamic cross‐linking network through acid‐base coupling between the C═O groups in TPU's hard segments and the Mn^2+^ of (ECMP)_2_MnBr_4_. This solvent‐mediated reversible dissociation mechanism (Figure 3c) yields exceptional elasticity while preserving the intrinsic mechanical properties of TPU. FTIR spectroscopic analysis provides direct evidence for this interfacial interaction (Figure 3d). The characteristic C═O stretching vibration at 1732 cm^−1^ in pristine TPU undergoes a 6 cm^−1^ redshift to 1726 cm^−1^ upon (ECMP)_2_MnBr_4_ incorporation, confirming the formation of coordination bonds between the polymer matrix and luminescent crystals. This molecular‐level interaction translates to macroscale mechanical enhancement, as demonstrated by tensile testing. Figure 3e demonstrates that the (ECMP)_2_MnBr_4_@ polydimethylsiloxane (thermoplastic polyurethane fibers demonstrate remarkable mechanical properties, sustaining strains exceeding 1000% while outperforming pristine TPU fibers in elasticity. This exceptional performance stems from the unique dynamic cross‐linking network formed between the luminescent crystals and polymer matrix, which not only preserves but significantly enhances the mechanical properties of the TPU framework. The exceptional deformability of the fibers is visually demonstrated in Figure 3f (with fiber diameters precisely maintained at 1.00±0.05 mm, as shown in Figure S13, Supporting Information). To validate practical applicability, we embroidered the luminescent fibers into an “SOS” pattern on everyday clothing (Figure 3g). Under daylight illumination, all letters remain visible, while upon 365 nm UV excitation, only the (ECMP)_2_MnBr_4_@TPU fibers emit intense green fluorescence, clearly displaying the predetermined emergency signal. Importantly, the fibers maintain stable luminescent performance even under various deformation modes (Figure 3h) and rub resistance (Video S1, Supporting Information), attributable to stress buffering by the porous TPU network, strong interfacial adhesion between (ECMP)_2_MnBr_4_ crystals and polymer matrix, and homogeneous elemental distribution preventing localized stress concentration. This study demonstrates the tremendous application potential of (ECMP)_2_MnBr_4_@TPU luminescent fibers in wearable emergency signaling systems, dynamic anti‐counterfeiting tags, flexible display devices, and smart textiles. Furthermore, we conducted a systematic mechanical durability assessment of the wet‐spun (ECMP)_2_MnBr_4_@TPU luminescent fibers. Quantitative analysis of the luminescence intensity variation under cyclic mechanical stresses (including repeated stretching, rubbing, and bending) reveals excellent stability, with negligible intensity degradation after 10 operational cycles, demonstrating the outstanding mechanical robustness of the composite fibers (Figure S12, Supporting Information).

**Figure 4:**
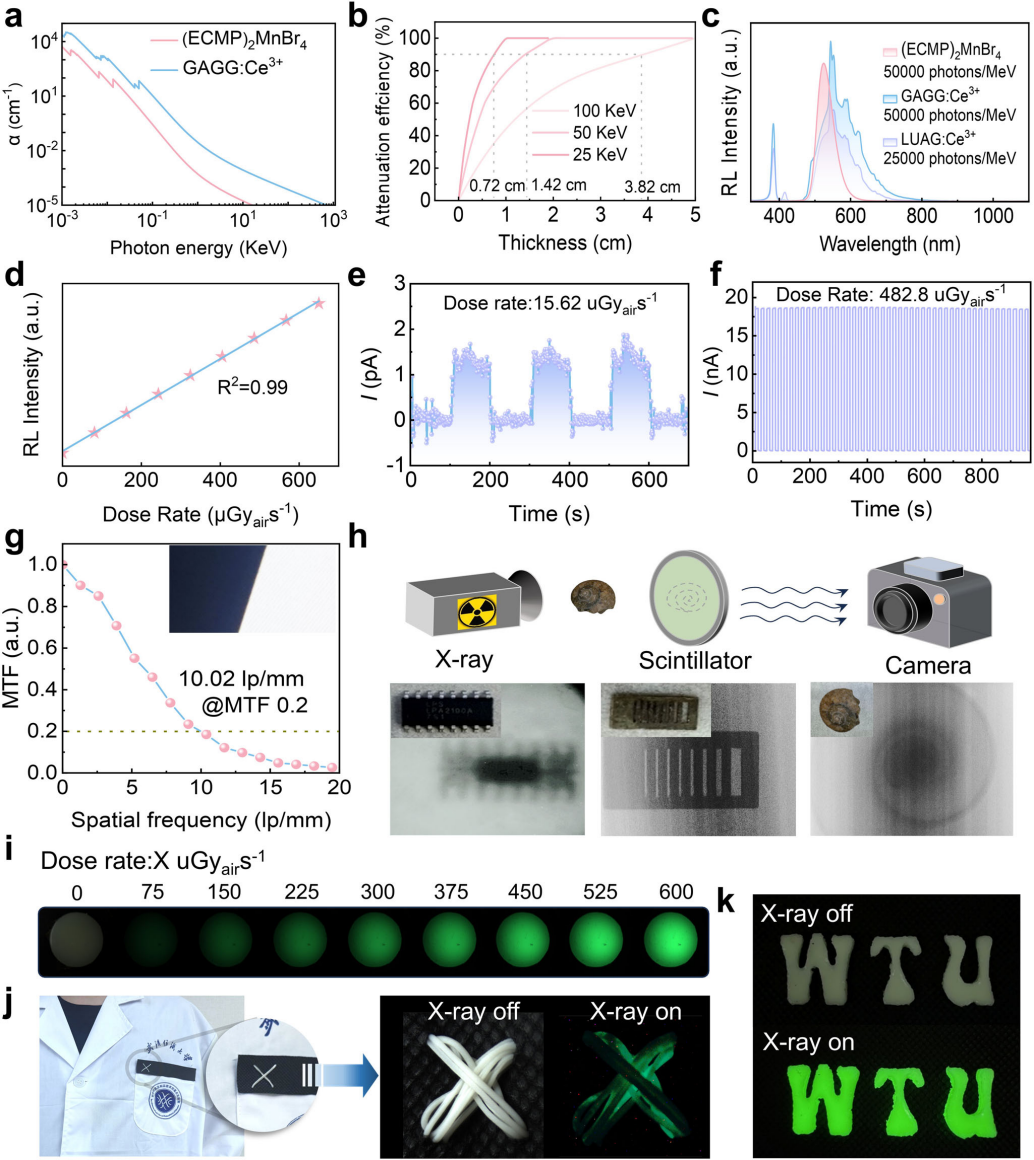
a) Comparative mass attenuation coefficients as a function of photon energy for (ECMP)_2_MnBr_4_ and commercial GAGG:Ce^3+^ scintillator, b) Calculated X‐ray attenuation efficiency of (ECMP)_2_MnBr_4_ at varying thicknesses, c) RL spectra and corresponding light yield comparison among (ECMP)_2_MnBr_4_, GAGG:Ce^3+^ and LUAG:Ce^3+^ scintillators, d) Linear response of RL intensity to X‐ray dose rate (50–650 µGy_air_ s^−1^) with correlation coefficient *R* ^2^> 0.99, e) X‐ray detection limit of (ECMP)_2_MnBr_4_, f) Radiation stability evaluated through over 50 on/off cycles at 482.8 µGy_air_ s^−1^, g) MTFs analysis quantifying spatial resolution (inset: X‐ray image of resolution test pattern), h) High‐resolution X‐ray imaging system (top) and corresponding X‐ray images (bottom) of semiconductor chip, test pattern, and snail sample, i) Dose‐dependent RL response of (ECMP)_2_MnBr_4_@PDMS films, j) Wearable integration showing (ECMP)_2_MnBr_4_@TPU fibers under visible light (left) and X‐ray irradiation (right), k) Patterned “WTU” letters fabricated from (ECMP)_2_MnBr_4_@PDMS composite under visible light (up) and X‐ray excitation (bottom).

Based on the exceptional luminescent properties of (ECMP)_2_MnBr_4_, we systematically investigated its potential for high‐resolution X‐ray imaging applications. Initial characterization involved calculating the attenuation coefficients of (ECMP)_2_MnBr_4_ crystals and commercial GAGG: Ce^3+^ scintillator across 0–1000 keV photon energies using the NIST XCOM database (**Figure** **4**a). Within the crucial imaging range (40–200 keV), (ECMP)_2_MnBr_4_ exhibited slightly lower attenuation coefficients than GAGG: Ce^3+^ due to its lower density. Subsequently, we investigated the attenuation efficiency of (ECMP)_2_MnBr_4_ at different photon energies (25, 50, 100 keV), the required thickness for 90% X‐ray attenuation demonstrated 3.82 cm at 100 keV, 1.42 cm at 50 keV, and merely 0.72 cm at 25 keV (Figure 4b), as determined by the attenuation law *η* = 1‐*e* ^−α*x*^, where α represents the linear attenuation coefficient and x denotes material thickness. This characteristic renders it particularly advantageous for lightweight detector design. Then, we measured the radioluminescence (RL) spectra of (ECMP)_2_MnBr_4_ crystals and commercial scintillators (GAGG: Ce^3+^ and LUAG: Ce^3+^) to compare their light yields. Remarkably, (ECMP)_2_MnBr_4_ exhibited a light yield of 50000 photons/MeV, comparable to GAGG: Ce^3+^ and twice that of LUAG: Ce^3+^ (25000 photons/MeV) (Figure 4c). Furthermore, we investigated the RL spectra of (ECMP)_2_MnBr_4_ crystals under varying X‐ray dose rates (Figure S14a, Supporting Information). The material demonstrated an excellent linear response (*R* ^2^ > 0.99) across a broad dose‐rate range (50–650 µGy_air_ s^−1^) (Figure 4d; Figure S14b, Supporting Information), indicating high sensitivity and precision in X‐ray detection. The detection limit is a critical parameter for evaluating scintillator performance, as a lower limit reduces radiation damage. Notably, (ECMP)_2_MnBr_4_ achieved an ultralow detection limit of 15.62 nGy_air_ s^−1^) in Figure 4e, which is three orders of magnitude lower than the medical diagnostic standard (5.5 µGy_air_ s^−1^).^[^ ^27^ ^]^ To assess practical utility, we conducted X‐ray irradiation stability tests on (ECMP)_2_MnBr_4_ crystals. After 50 on‐off irradiation cycles, the material retained > 96% of its initial RL intensity (Figure 4f), confirming exceptional radiation resistance. We constructed a simple imaging system and successfully captured high‐resolution X‐ray images (Figure 4h, top). The spatial resolution was quantitatively evaluated using the slanted‐edge method, yielding a modulation transfer function (MTF) curve (Figure 4g). The scintillator film achieved a resolution of 10.02 lp mm^−1^ at MTF = 0.2 (Note S2, Supporting Information), making it highly attractive for imaging fine microstructures. Leveraging this system, we obtained clear X‐ray images (X‐ray dose rate, 150 µGy_air_ s^−1^) of a semiconductor chip, a test pattern, and a snail specimen (Figure 4h, bottom), vividly revealing their internal micron‐scale features. To explore broader applications, we fabricated (ECMP)_2_MnBr_4_@polydimethylsiloxane (PDMS) composite films (1:2 mass ratio) and examined their RL intensity under X‐ray doses ranging from 0 to 600 µGy_air_ s^−1^) (Figure 4i). The RL intensity increased linearly with dose rate, consistent with the results in Figure 4d. Additionally, we integrated wet‐spun (ECMP)_2_MnBr_4_@TPU fibers onto a lab coat (Figure 4j) and patterned a “WTU” logo using (ECMP)_2_MnBr_4_@PDMS (Figure 4k). Both configurations emitted bright green light under X‐ray excitation, matching the performance of single crystals. These results underscore the material's potential for portable radiation detectors, wearable protective gear, and real‐time radiation visualization systems.

**Figure 5:**
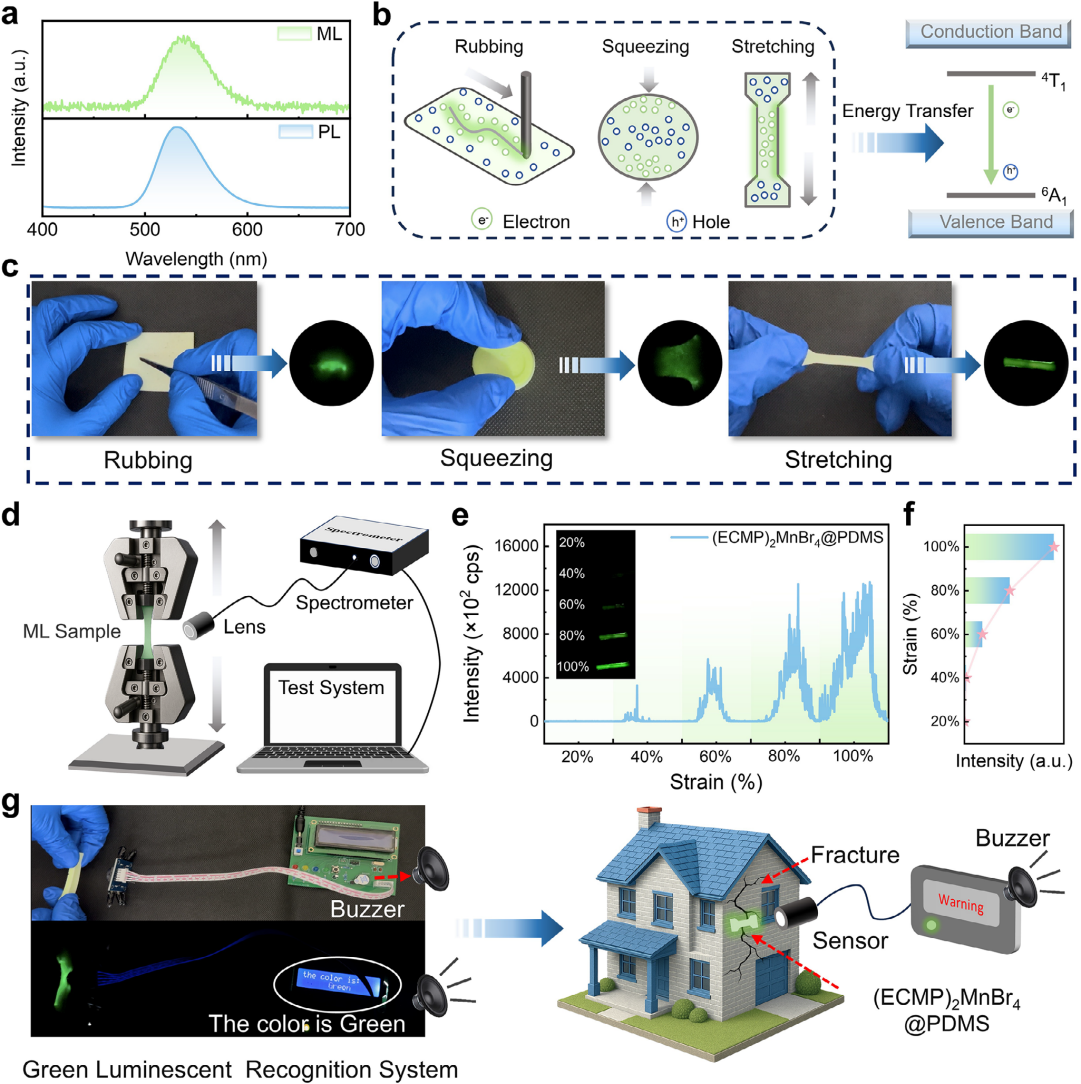
a) Spectral comparison between ML and PL emissions from (ECMP)_2_MnBr_4_, b) Three mechanical excitation modalities (rubbing, squeezing, and stretching) applied to (ECMP)_2_MnBr_4_@PDMS elastomers, c) Time‐resolved ML imaging of composite films under mechanical stimuli corresponding to panel (b), d) Experimental setup for ML spectral measurements, e) Strain‐dependent ML spectral evolution, f) Quantitative comparison of integrated ML intensity versus applied tensile stress, g) Proof‐of‐concept demonstration of an ML‐based hazard warning system utilizing (ECMP)_2_MnBr_4_’s stress‐responsive luminescence.

Furthermore, we also systematically investigated the mechanoluminescence (ML) characteristics of (ECMP)_2_MnBr_4_, which was uniformly dispersed in the PDMS matrix (Figure S15, Supporting Information). Spectral characterization revealed identical emission peaks at 530 nm for both ML and PL spectra (**Figure** **5**a), with this spectral congruence unequivocally establishing that the ML emission stems from the same Mn^2+ 4^T_1_→^6^A_1_ transition as the PL. This observation indicates a shared excited‐state origin despite distinct excitation pathways. As evidenced in Figure 5b, mechanical stimulation (rubbing, squeezing, or stretching) of (ECMP)_2_MnBr_4_ crystals induces rupture of the hydrogen‐bonding network, generating localized charge separation at fracture interfaces. The resultant electric fields accelerate electrons that non‐radiatively transfer energy to the Mn^2+^ centers, ultimately producing efficient ML via the ^4^T_1_→^6^A_1_ transition. These mechanistic insights provide fundamental guidance for subsequent experimental investigations. As demonstrated in Figure 5c and Video S2 (Supporting Information), the (ECMP)_2_MnBr_4_@PDMS films exhibited bright green emission under various mechanical stimuli. Quantitative evaluation using a spectromechanical testing system (Figure 5d) showed that the ML intensity increased linearly with applied stretching within the 20–100% stretching range (Figure 5e,f). This excellent stress‐luminescence linearity suggests the material's potential as an ideal mechanical sensor. Leveraging these properties, we developed a novel mechano‐responsive warning system (Figure 5g). When subjected to critical stress, the film's visible green emission triggers optoelectronic sensors to activate audible/visual alarms. This system offers unique advantages, self‐powered operation without an external power supply, sub‐second response time, and excellent reusability. Particularly in health monitoring applications, this material enables real‐time visualization of stress distribution, providing intuitive optical signals for disaster warning (e.g., collapse, crack propagation), demonstrating significant engineering value.

The recyclability of (ECMP)_2_MnBr_4_ was systematically demonstrated through a reversible crystallization‐dissolution process in Figure S16a (Supporting Information). When maintaining the precursor solution at 70 °C, high‐quality single crystals with intense green emission under UV illumination were obtained. Subsequent heating to 100 °C induced complete dissolution, while recooling to 70 °C reproducibly yielded crystals with identical morphological and luminescent properties, confirming excellent recyclability over five consecutive recycling cycles. Remarkably, (ECMP)_2_MnBr_4_@PDMS composites exhibited exceptional environmental stability when subjected to accelerated aging tests. The organic ECMP⁺ cations form both *π–π* stacking interactions and hydrogen‐bonding networks with the inorganic framework. This dual interaction mechanism maintains structural integrity while suppressing non‐radiative decay pathways, thereby significantly enhancing the material's environmental stability. After 49 days of exposure to ambient atmosphere (Figure S16b, Supporting Information), acidic (pH 1, Figure S16c, Supporting Information), and alkaline (pH 14, Figure S16d, Supporting Information) conditions, PL spectra revealed maintained emission intensity without significant degradation. While minor intensity fluctuations were observed‐likely due to surface adsorption phenomena‐the absence of any systematic decreasing trend underscores the material's robustness for applications in harsh environments. This unique combination of recyclability and environmental stability positions (ECMP)_2_MnBr_4_ as an outstanding candidate for sustainable optoelectronic applications, particularly where material recovery and durability under variable conditions are paramount.

## 3 Conclusion

This study successfully synthesized 0D organic–inorganic hybrid manganese halide single crystals, (ECMP)_2_MnBr_4_, via an eco‐friendly low‐temperature crystallization method (ethanol solvent, 70 °C), the synergistic combination of rigid [MnBr_4_]^2−^ tetrahedra and dynamic ECMP⁺ *π–π* stacking enables unprecedented triple‐mode emission (98.97% PLQY, 15.62 nGy_air_ s^−1^ X‐ray detection limit and multi‐stimuli responsiveness) while resolving the performance‐wearability trade‐off. Our breakthrough wet‐spinning technique yields (ECMP)_2_MnBr_4_@TPU fibers that maintain single‐crystal emission fidelity with exceptional mechanical robustness (>1000% strain), representing the first successful integration of 0D Mn (II) emitters into elastomeric matrices. The material system exhibits complete recyclability and unprecedented environmental stability (>90% PL retention in pH 1–14), achieved through an integrated “molecular design‐crystal engineering‐macroscopic integration” approach. Demonstrated applications in medical‐grade radiation monitoring, deformation‐resistant optical encryption, and self‐powered hazard detection validate its versatility, while the established design principles provide a universal framework for developing next‐generation smart materials that combine atomic‐level coordination control with scalable manufacturing. These advances create new opportunities for sustainable, multifunctional optoelectronic systems across wearable electronics, environmental sensing, and biomedical diagnostics, with the potential to extend beyond manganese halides to other hybrid material systems.

## Conflict of Interest

The authors declare no conflict of interest.

## Supporting information



Supporting Information

Supplemental Video 1

Supplemental Video 2

## Data Availability

The data that support the findings of this study are available from the corresponding author upon reasonable request.
