# Supplementary material for: Green Synthesis and Flexibilization Engineering of (ECMP)2MnBr4 for Smart Textile‐Integrated Luminescence
Source: Adv Sci (Weinh). 2025 Aug 31;12(42):e11652. doi: 10.1002/advs.202511652 (PMC12622510; doi:10.1002/advs.202511652)
Supplement: Supplementary file 1 — Supporting Information [file ADVS-12-e11652-s001.pdf]

Green Synthesis and Flexibilization Engineering of $(ECMP)_2MnBr_4$ for Smart Textile-Integrated Luminescence

Xiao Wang[#,1], Yanyan Li[#,*1], Haitao Tang[2], Wenxuan Yao[3], Jiao Li[3], Fang Yao[1], Weibing Zhong[1], Kangyu Jia[1], Dong Tu[3], Qianqian Lin[*2], Mufang Li[1], Dong Wang[*1]

[1] Key Laboratory of Textile Fiber and Products, Wuhan Textile University, Ministry of Education, Wuhan 430200, China

[2] School of Physics and Technology, Wuhan University, Wuhan 430200, China

[3] Faculty of Materials Science and Chemistry, China University of Geosciences, Wuhan 430074, China.

E-mail address: liyy@wtu.edu.cn (Y. Li), q.lin@whu.edu.cn (Q. Lin), wangd@wtu.edu.cn (D. Wang)

Keywords: 0D perovskites, green synthesis, manganese bromides, smart textiles

Abstract

0D hybrid manganese halides represent an emerging class of luminescent materials, yet their practical application has been hindered by the intrinsic trade-off between optical performance and mechanical flexibility. Here, we report a green-synthesized 0D $(ECMP)_2MnBr_4$ crystal exhibiting unprecedented triple-mode emission (photoluminescence, X-ray scintillation, and mechano cluminescence) through rationally designed highly symmetric $[MnBr_4]^{2-}$ tetrahedra, achieving near-unity photoluminescence quantum yield (98.97%), record-low X-ray detection limit (15.62 nGy_{air} s^{-1}) and multi-stimuli responsiveness (rubbing, squeezing, stretching). The material's ultralow electron-phonon coupling (S=1.438) and defect-suppressing π-π stacking enable exceptional environmental stability and closed-loop recyclability via solvent-mediated recrystallization. Innovatively, we first integrate $(ECMP)_2MnBr_4$ into thermoplastic polyurethane via wet-spinning, simultaneously retaining single-crystal emission intensity and achieving remarkable elasticity (>1000% strain) for deformation-resistant wearable applications. This work establishes a new design

paradigm for sustainable multifunctional optoelectronics, with immediate applications in wearable displays, high-resolution X-ray imaging, and self-powered optical sensors.

Contents

1. Experimental details 4

2. Supporting notes 5

3. Supporting figures 6

1. Experimental details

1.1 Materials

Ethoxycarbonylmethyl(triphenyl)phosphonium bromide (ECMPBr, 98%) was purchased from Shanghai Macklin Biochemical Corporation. Manganese (II) bromide ($MnBr_2$, 99.99%) and Ethyltriphenylphosphonium bromide (ETPBr, 98%) were purchased from Aladdin Industrial Corporation. Ethyl alcohol (EtOH, 99%) was acquired from Sinopharm Chemical Reagent Company. All commercial products were used as received.

1.2 Preparation of $(ECMP)_2MnBr_4$, $(ETP)_2MnBr_4$, $(ECMP)_2MnBr_4$@TPU spinning solution and $(ECMP)_2MnBr_4$@PDMS

$(ECMP)_2MnBr_4$ and $(ETP)_2MnBr_4$ luminescent crystals were synthesized by solution method, and the dosage was strictly weighed according to stoichiometric ratio.

Preparation of $(ECMP)_2MnBr_4$: 2 mmol ECMPBr and 1 mmol $MnBr_2$ were mixed in a reagent bottle with 5 mL EtOH. Stir the mixture and dissolve completely in a fume hood at 100°C. Then, cool down to 70 °C and stop stirring, precipitate the crystals. Finally, the product is removed from the reactor and then dried at 60°C.

Preparation of $(ETP)_2MnBr_4$: ECMPBr is substituted by ETPBr according to the molar ratio, the $(ETP)_2MnBr_4$ synthesis processes are consistent with the steps for $(ECMP)_2MnBr_4$.

Preparation of $(ECMP)_2MnBr_4$@TPU spinning solution: Mix ECMPBr, $MnBr_2$, and TPU particles in N, N-Dimethylformamide (DMF) solvent (mmol ECMPBr: $MnBr_2$: TPU=2:1:2, m TPU: DMF=2:8), and stir on a stir plate for 12 hours to obtain the spinning solution.

Preparation of $(ECMP)_2MnBr_4$@TPU fiber: Homogeneous spinning solution was prepared by dissolving ECMPBr, $MnBr_2$, and TPU particles in N, N-dimethylformamide (DMF) under stoichiometric control (ECMPBr: $MnBr_2$: TPU =

2:1:2 molar ratio; TPU: DMF = 2:8 mass ratio) with continuous stirring for 12 h at room temperature. The resulting solution was loaded into a syringe equipped with a 19G needle and infused into a deionized water coagulation bath (room temperature) at a controlled injection rate of 0.5 mL/min using an electrospinning apparatus. After immersion for 30 min to ensure complete phase separation, the solidified fibers were carefully extracted, air-dried in a fume hood, and stored under ambient conditions.

Preparation of $(ECMP)_2MnBr_4$@PDMS: ECMPBr and $MnBr_2$ were mixed with polydimethylsiloxane (PDMS) at molar ratio of 2:1 (ECMPBr: $MnBr_2$) and mass ratio of 2:1 ($(ECMP)_2MnBr_4$: PDMS), followed by drying at 60°C in an oven.

1.3 Characterizations

The crystal structure was evaluated with X-ray diffraction (XRD, D8 Advance X-ray diffractometer) with a scanning range from 0 to 80°. Steady-state photoluminescence (PL) spectra were recorded using a Morpho Nova spectrometer under the excitation of a 362 nm CW laser. Morphology of crystals was examined by scanning electron microscope (SEM, TESCAN MIRA3). The absorption spectra of crystals were measured using a spectrophotometer (PerkinElmer LAMBDA 1050). The attenuation curve and quantum yield were recorded with a photoluminescence spectrometer (FLS1000). X-ray photoelectron spectroscopy (XPS) measurements were conducted using an XPS system (Thermo Scientific, ESCALAB 250Xi).

2. Supporting notes

Note 1: First principles

We perform first-principles calculations based on density functional theory (DFT)[1], as implemented in the GPU-accelerated PWmat package[2, 3]. Specifically, PWmat is employed to compute the structural, electronic, and defect properties of three distinct systems. For the exchange-correlation potential, we adopt the generalized gradient approximation (GGA) in the form of the Perdew-Burke-Ernzerhof (PBE) functional[4, 5].

Structural relaxations are carried out with a force convergence criterion of 0.02 eV/Å for the maximum residual force.

For band structure, density of states, and charge density difference calculations, we utilize the Heyd-Scuseria-Ernzerhof (HSE06) hybrid functional[6], incorporating a Fock exchange parameter (α=0.25) and a screening parameter (ω=0.2Å^{-1})[3]. Geometry optimizations are performed using a Monkhorst-Pack k-point mesh with a spacing of 0.04 Å^{-1}. All calculations are conducted within the PWmat framework using norm-conserving pseudopotentials[7] and a plane-wave cutoff energy of 60 Rydberg.

Note 2: MTF measurements

The modulation transfer function (MTF) quantitatively characterizes the spatial resolution performance of an imaging system by describing its ability to maintain signal modulation across varying spatial frequencies. An ideal system would exhibit an MTF value of 1.0, representing perfect signal transfer at a given spatial frequency. For our measurements, we employed the standardized slanted-edge methodology. The experimental procedure involved: 1. Imaging a precisely machined aluminum edge (thickness: 1.0 ± 0.1 mm) under X-ray irradiation; 2. Extracting the edge spread function (ESF) from the obtained edge profile; 3. Computing the line spread function (LSF) through numerical differentiation of the ESF; 4. Determining the MTF curve via discrete Fourier transformation of the LSF. The MTF curves could be calculated as,

$$MTF(v) = F\big(LSF(x)\big) = F\frac{\mathrm{d}ESF(\mathrm{x})}{dx}$$

where v represents spatial frequency, x denotes the position of pixels. Due to using different optical system, the position of pixels is defined as follows:

$$x = \frac{\mathrm{N.d}}{\beta}$$

where N represents the ordinal number of pixels in X-ray edge image, d denotes the pixel pitch (11 μm) and β corresponds to the optical magnification.

3. Supporting figures and tables

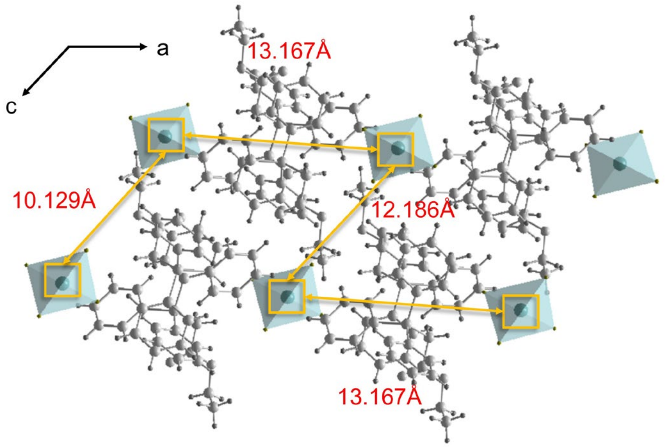

Figure S1. Crystal structure of $(ECMP)_2MnBr_4$, showing the distances between adjacent Mn^{2+} ions. The longest and shortest distances between adjacent Mn^{2+} ions are 13.167 Å and 10.129 Å, respectively.

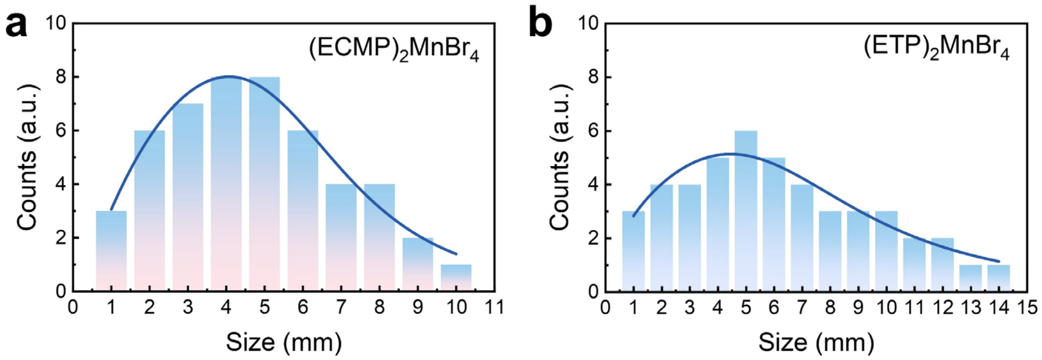

Figure S2. Size distributions of (a) $(ECMP)_2MnBr_4$ and (b) $(ETP)_2MnBr_4$.

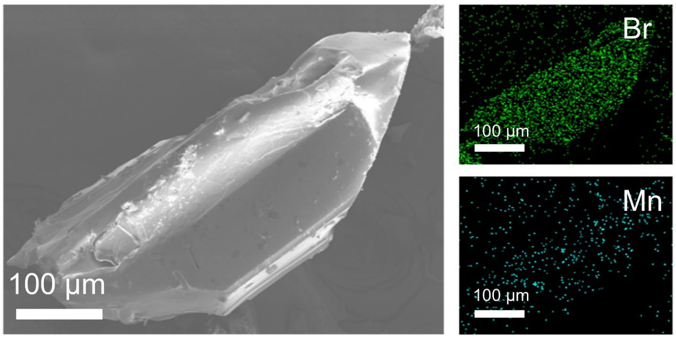

Figure S3. SEM image and corresponding EDS elemental mapping of $(ETP)_2MnBr_4$.

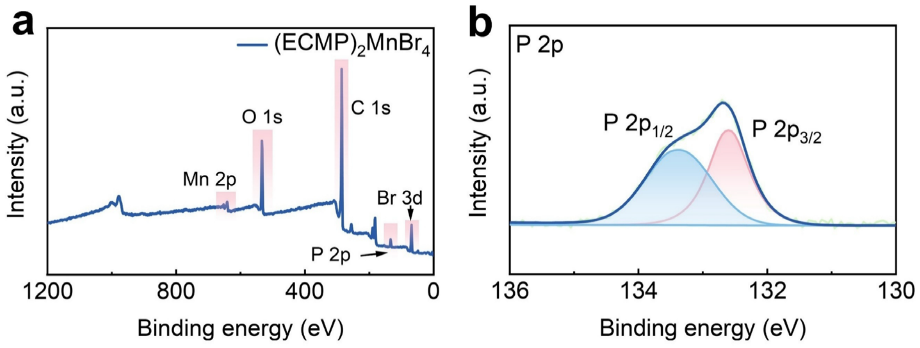

Figure S4. XPS analysis of $(ECMP)_2MnBr_4$. (a) Full survey spectrum, (b) High-resolution spectrum of the P 2p core-level region.

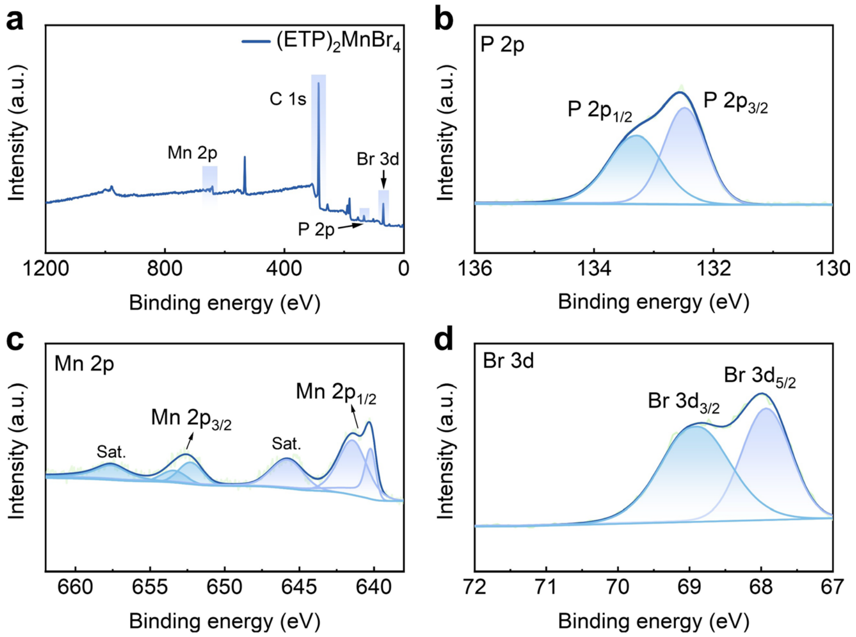

Figure S5. XPS analysis of $(ETP)_2MnBr_4$. (a) Full survey spectrum, (b) High-resolution spectrum of the P 2p, (c) High-resolution spectrum of the Mn 2p, (d) High-resolution spectrum of the Br 3d.

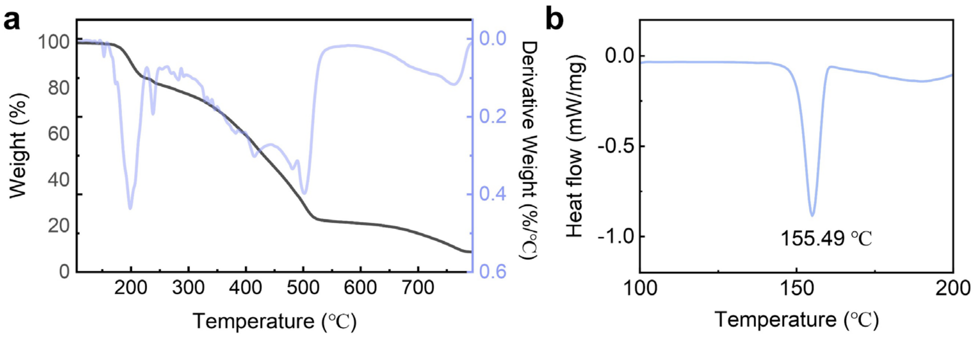

Figure S6. (a) Thermogravimetric analysis curve of $(ECMP)_2MnBr_4$, (b) Differential

scanning calorimetry curve of $(ECMP)_2MnBr_4$.

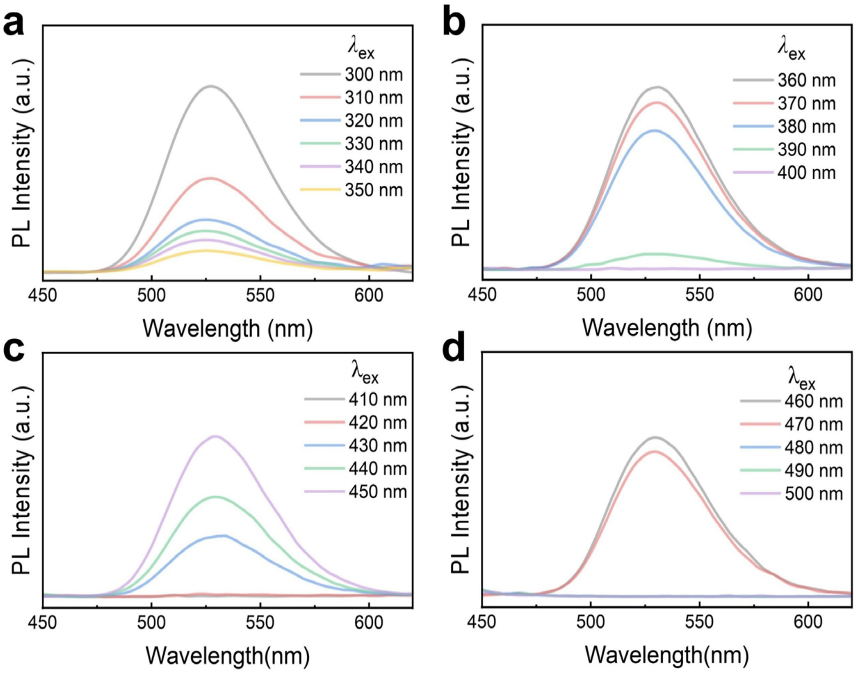

Figure S7. PL spectra of $(ECMP)_2MnBr_4$ recorded at varying excitation wavelengths.

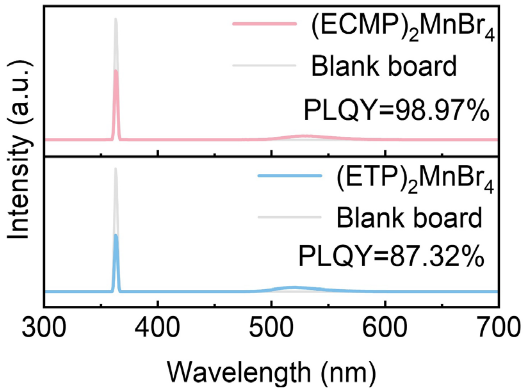

Figure S8. Comparative PLQY measurements of $(ECMP)_2MnBr_4$ and $(ETP)_2MnBr_4$.

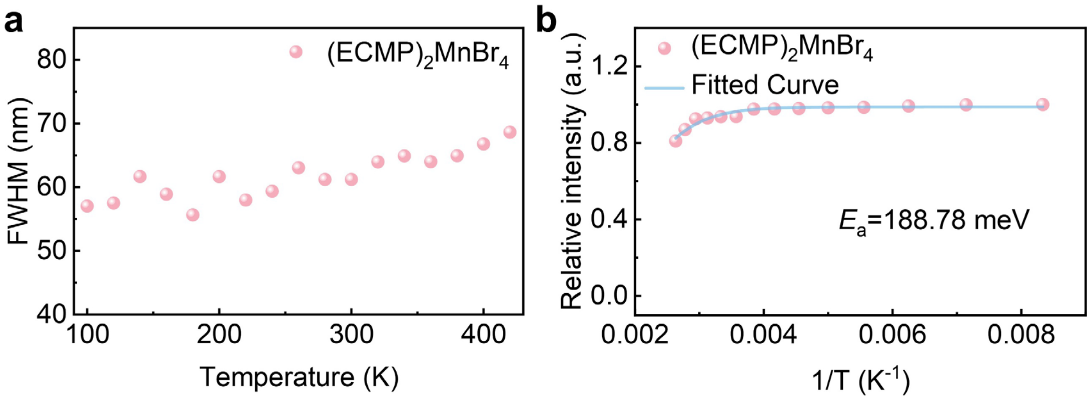

Figure S9. (a) Temperature-dependent FWHM of the $(ECMP)_2MnBr_4$, (b) Arrhenius

fitting of the temperature dependent PL intensity of $(ECMP)_2MnBr_4$.

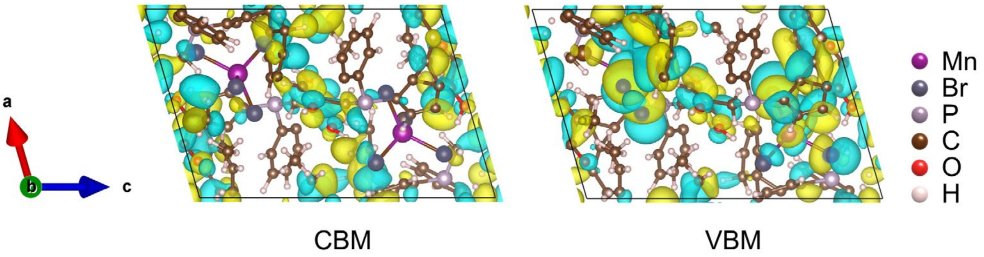

Figure S10. Charge distribution for $(ECMP)_2MnBr_4$ that corresponds to the CBM (left) and VBM (right).

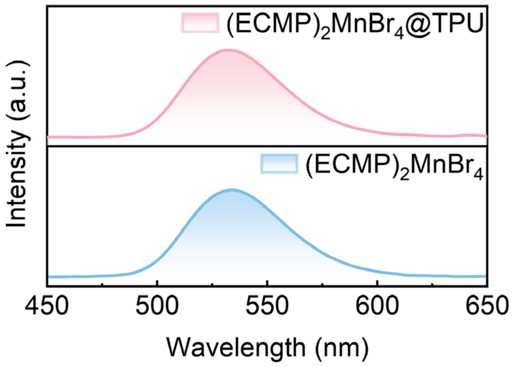

Figure S11. PL emission spectra of $(ECMP)_2MnBr_4$@TPU and pristine $(ECMP)_2MnBr_4$.

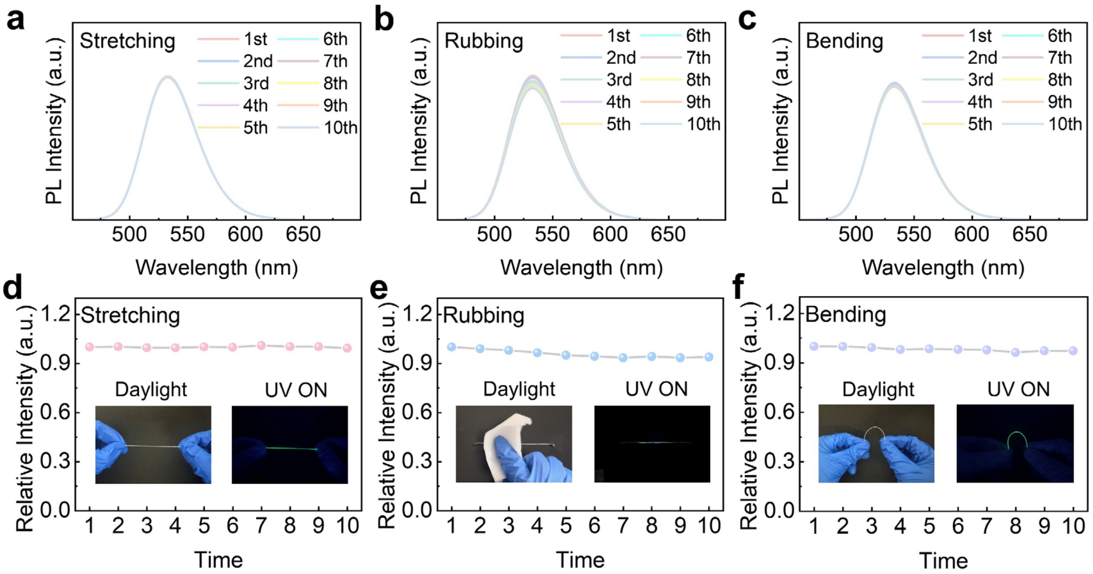

Figure S12. Luminescence intensity versus number of mechanical cycles for evaluating the mechanical durability of wet-spun fibers: (a, d) Stretching, (b, e) Rubbing, and (c,

f) Bending.

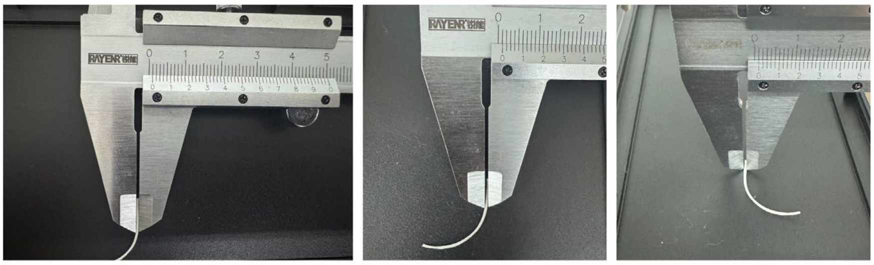

Figure S13. Diameter measurements of $(ECMP)_2MnBr_4$@TPU wet-spun fibers obtained using a digital vernier caliper.

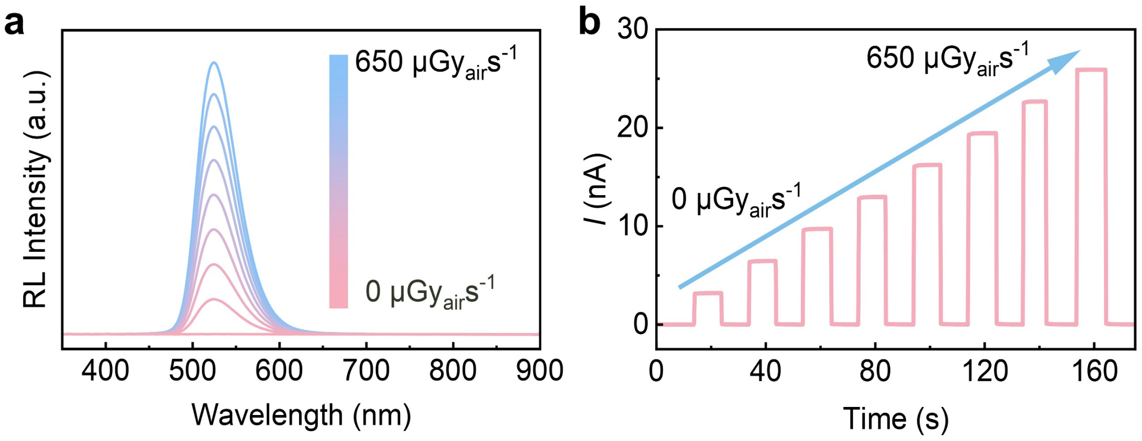

Figure S14. (a) RL spectra of $(ECMP)_2MnBr_4$ recorded under X-ray irradiation with dose rates ranging from 0 to 650 $\mu Gy_{air}s^{-1}$, (b) Dose-rate-dependent X-ray response characteristics of $(ECMP)_2MnBr_4$.

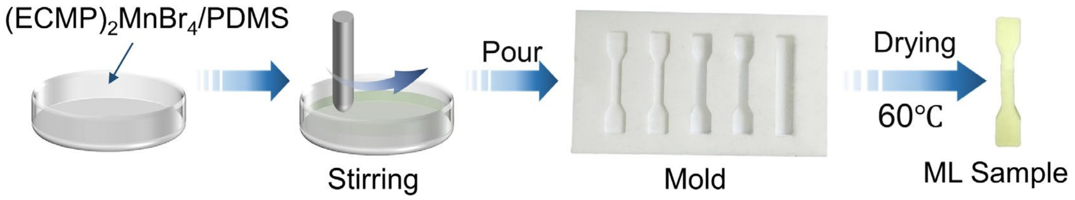

Figure S15. Schematic illustration of the synthesis process for mechanoluminescent $(ECMP)_2MnBr_4$@TPU.

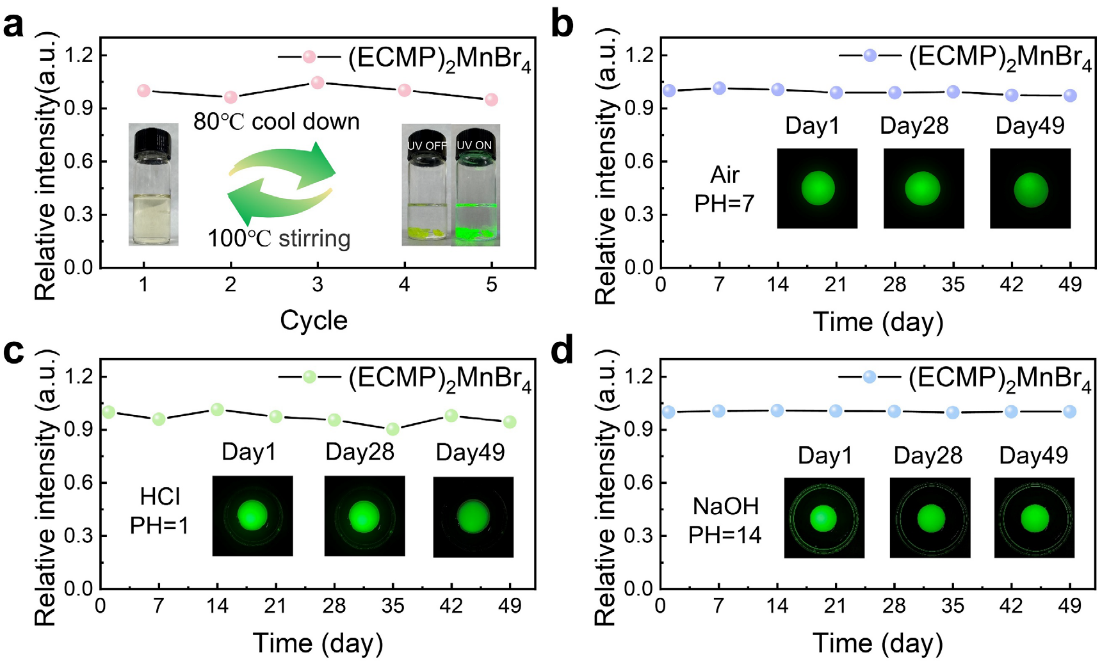

Figure S16. Schematic illustration of $(ECMP)_2MnBr_4$ recyclability and stability assessment. (a) Recyclability, PL intensity retention of $(ECMP)_2MnBr_4$ under various environmental conditions of (b) Ambient atmosphere, (c) Acidic solution (HCl, pH=1), (d) Alkaline solution (NaOH, pH=14).

Table S1. Crystal structure parameters of $(ECMP)_2MnBr_4$.

Chemical formula	$(C_{22}H_{22}O_2P)_2MnBr_4$
Mr	1073.31
Crystal system	Monoclinic
Space group	*C*2/*c*
Temperature (K)	193
a (Å)	23.341 (2)
b (Å)	12.1948 (11)
c (Å)	18.415 (3)
α (deg)	90
β (deg)	121.922(3)

γ (deg)	90
V (Å^3)	4449.0 (10)
Z	4
Density (g cm^{-3})	1.602
F (000)	2140
μ (mm^{-1})	4.00
R [$F^2 > 2\sigma(F^2)$]	0.041
wR (F^2)	0.098
GOF on F^2 (S)	1.03

Table S2. Selected bond lengths of $(ECMP)_2MnBr_4$.

Bond	Length (Å)
Br2 - Mn1	2.4937 (6)
Br1 - Mn1	2.5092 (6)

Table S3. Selected bond angles of $(ECMP)_2MnBr_4$.

Bond	Angle (°)
$Br2^{i}$ - Mn1 - Br2	108.20 (3)
Br2 - Mn1 - Br1	104.000 (14)
$Br2^{i}$ - Mn1 - $Br1^{i}$	104.000 (14)
$Br2^{i}$ - Mn1 - Br1	115.898 (15)
Br2 - Mn1 - $Br1^{i}$	115.900 (15)

Br1 - Mn1 - Br1^{i}	109.27 (4)

Table S4. Photoluminescence lifetime (τ) comparison of $(ECMP)_2MnBr_4$ and $(ETP)_2MnBr_4$

Scintillators	τ_1(ms)	τ_2(ms)	τ_{avg}(ms)	R^2
$(ECMP)_2MnBr_4$	3.2728	3.2729	3.272	0.998
$(ETP)_2MnBr_4$	3.0978	3.1008	3.099	0.998

Table S5. Comparison of X-ray detection limits based on typical metal halides

Scintillators	Limit of Detection ($nGy_{air}\ s^{-1}$)	Ref.
$(C_{24}H_{20}P)_2MnBr_4$	608	[8]
$(BPTP)_2MnBr_4$	282	[9]
$(HTPP)_2MnBr_4$	185	[10]
$(C_{25}H_{22}P)_2MnBr_4$	108.2	[11]
$(ETP)_2MnBr_4$	103.1	[12]
$(MTP)_2MnBr_4$	82.4	[13]
$(C_{38}H_{34}P_2)MnBr_4$	72.8	[14]
$(TBA)_2MnBr_4$	63.3	[15]
$(ECMP)_2MnBr_4$	15.62	This work

$C_{24}H_{20}P^+$=tetraphenylphosphonium,

$BPTP^+$=(3-bromopropyl)triphenylphosphonium,

$HTPP^+$=hexyltriphenylphosphonium,

$C_{25}H_{22}P^+$=benzyltriphenylphosphonium,

ETP^+=ethyltriphenylphosphonium,

MTP^+=ethyltriphenylphosphonium,

$C_{38}H_{34}P_2^+$=ethyl-enebistriphenylphosphonium,

TBA^+=tetrabutylammonium,

$ECMP^+$=(ethoxycarbonyl-methyl)triphenylphosphonium.

References

1. W. Kohn and L. J. Sham, *Phys. Rev.*, **1965**, *140*, A1133-A1138.
2. W. Jia, J. Fu, Z. Cao, L. Wang, X. Chi, W. Gao and L. W. Wang, *J. Comput. Phys.*, **2013**, *251*, 102-115.
3. A. V. Krukau, O. A. Vydrov, A. F. Izmaylov and G. E. Scuseria, *J. Chem. Phys.*, **2006**, *125*, 224106.
4. J. P. Perdew, K. Burke and M. Ernzerhof, *Phys. Rev. Lett.*, **1996**, *77*, 3865-3868.
5. P. Ziesche, S. Kurth and J. P. Perdew, *Comput. Mater. Sci.*, **1998**, *11*, 122-127.
6. J. Heyd, G. E. Scuseria and M. Ernzerhof, *J. Chem. Phys.*, **2003**, *118*, 8207-8215.
7. H. J. Monkhorst and J. D. Pack, *Phys. Rev. B*, **1976**, *13*, 5188-5192.
8. K. Xia, P. Ran, W. Wang, J. Yu, G. Xu, K. Wang, X. Pi, Q. He, Y. Yang and J. Pan, *Adv. Opt. Mater.*, **2022**, *10*, 2201028.
9. R. Zhang, H. Xie, W. Liu, K. Zhan, H. Liu, Z. Tang and C. Yang, *ACS Appl. Mater. Interfaces*, **2023**, *15*, 47238-47249.
10. J. B. Luo, J. H. Wei, Z. Z. Zhang, Z. L. He and D. B. Kuang, *Angew. Chem. Int. Ed.*, **2023**, *62*, 202216504.
11. M. Zhou, H. Jiang, T. Hou, S. Hou, J. Li, X. Chen, C. Di, J. Xiao, H. Li and D. Ju, *Chem. Eng. J.*, **2024**, *490*, 151823.
12. B. Li, Y. Xu, X. Zhang, K. Han, J. Jin and Z. Xia, *Adv. Opt. Mater.*, **2022**, *10*, 2102793.
13. W. Zhang, P. Sui, W. Zheng, L. Li, S. Wang, P. Huang, W. Zhang, Q. Zhang, Y. Yu and X. Chen, *Angew. Chem. Int. Ed.*, **2023**, *62*, 202309230.
14. L. J. Xu, X. Lin, Q. He, M. Worku and B. Ma, *Nat. Commun.*, **2020**, *11*, 4329.
15. W. Ma, D. Liang, Q. Qian, Q. Mo, S. Zhao, W. Cai, J. Chen and Z. Zang, *eScience*, **2023**, *3*, 100089.
